# Autophagy in the physiology and pathology of the central nervous system

**DOI:** 10.1038/cdd.2014.204

**Published:** 2014-12-19

**Authors:** V Nikoletopoulou, M-E Papandreou, N Tavernarakis

**Affiliations:** 1 Institute of Molecular Biology and Biotechnology, Foundation for Research and Technology-Hellas, Heraklion, Crete 71110, Greece; 2 Department of Basic Sciences, Faculty of Medicine, University of Crete, Heraklion, Crete 71110, Greece

## Abstract

Neurons are highly specialized postmitotic cells that depend on dynamic cellular processes for their proper function.These include among others, neuronal growth and maturation, axonal migration, synapse formation and elimination, all requiring continuous protein synthesis and degradation. Therefore quality-control processes in neurons are directly linked to their physiology. Autophagy is a tightly regulated cellular degradation pathway by which defective or superfluouscytosolic proteins, organelles and other cellular constituents are sequestered in autophagosomes and delivered to lysosomes for degradation. Here we present emerging evidence indicating that constitutive autophagic fluxin neurons has essential roles in key neuronal processes under physiological conditions.Moreover, we discuss how perturbations of the autophagic pathway may underlie diverse pathological phenotypes in neurons associated with neurodevelopmental and neurodegenerative diseases.

## Facts

- Autophagy occurs constitutively in neurons under physiological conditions.
- Impairment of constitutive autophagy leads to neurodegeneration.
- Autophagic flux in the retinal pigmented epithelium (RPE) is critical for the visual cycle.
- Autophagy-regulated lipid metabolism within hypothalamic neurons modulates neuropeptide levels.
- Impaired autophagy has been implicated in the pathogenesis of many neurodevelopmental and neurodegenerative disorders.

## Open Questions

- How does deficiency in specific types of macroautophagy, such as mitophagy, nucleophagy, lipophagy and so on, impact neuron physiology?
- Does glial-specific deficiency in macroautophagy affect the physiology of the brain? If yes, how?
- Is long-term potentiation (LTP) and/or long-term depression affected by manipulating autophagic flux levels?
- How is constitutive autophagy regulated in healthy neurons?
- How does chronic autophagy disruption affect neuronal circuitry and other complex brain processes?
- Does pharmacological intervention inducing selective types of macroautophagy halt progression of disease?
- Development of tools to image and monitor selective types of autophagy in the central nervous system (CNS) *in vivo* is essential.
- Identification of the molecular mechanisms of autophagy modulation under diverse conditions is critical for the development of therapeutic drugs.

**Figure 1:**
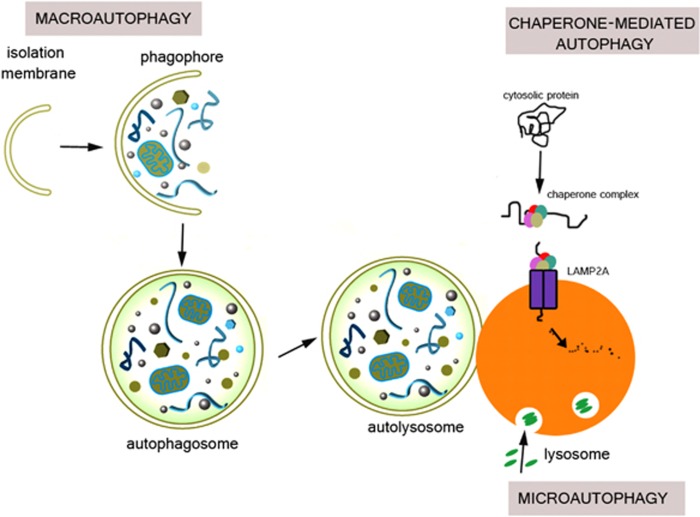
Schematic representation of the three types of autophagy: Microautophagy, CMA, and macroautophagy. In microautophagy, invaginations of the lysosomal membrane directly engulf portions of the cytoplasm. By contrast, CMA involves the chaperone Hsc70 and its co-chaperones that recognize and unfold substrate proteins and then bind to the lysosomal protein LAMP2A and are translocated across the lysosomal membrane for degradation. In macroautophagy, substrates are sequestered by an isolation membrane (known as the phagophore), which elongates and eventually seals to surround the substrate, forming a double membranous structure, the autophagosome. Autophagosomes then fuse with the lysosome to form autolysosomes

Cells require a continuous recycling of their cytoplasm to generate macromolecular building blocks and energy both under physiological and stress conditions. Autophagy (from Greek, meaning self-eating) is a regulated process for the bulk degradation of cytosolic components and organelles through delivery to lysosomes. In addition, autophagy facilitates the removal of superfluous and damaged organelles to help cells adapt to changing nutrient conditions and maintain their homeostasis. Autophagy also has a critical role in cytoprotection by preventing the accumulation of toxic proteins and through its action in various aspects of immunity, including the elimination of invasive microbes and its participation in antigen presentation.^1^ There are three distinct classes of autophagy, as summarized in Figure 1: microautophagy, chaperone-mediated autophagy (CMA), and macroautophagy.^2^ In microautophagy, invaginations of the lysosomal membrane directly engulf portions of the cytoplasm. By contrast, CMA involves the chaperone HSC70 and its co-chaperones that recognize and unfold substrate proteins with a KFERQ amino-acid motif. These substrates bind to the lysosomal protein LAMP-2A and are translocated across the lysosomal membrane for degradation.^3^

Macroautophagy is the major type of autophagic process and differs from the other two types in that the substrates are sequestered by anisolation membrane (known as the phagophore), which elongates and eventually seals to surround the substrate, forming a double membranous structure, the autophagosome.The autophagosome then fuses with the lysosome to form an autolysosome, in which the hydrolytic degradation of contents of the autophagosome occurs. In addition to cytosolic proteins, the substrates of macroautophagy also include superfluous and damaged organelles, such as mitochondria, peroxisomes and nuclei, lipids and invasive microbes. These selective types of macroautophagy are termed mitophagy, pexophagy, nucleophagy and lipophagy for mitochondria, peroxisomes, nuclei and lipids, respectively.We will focus our discussion on macroautophagy, herein referred to simply as autophagy.

## Mechanistic Aspects of Autophagy

Understanding of the molecular pathway of autophagy has been achieved by identifying several autophagy genes (ATG) conserved from yeast to mammals. Mechanistically, autophagy can be broken down into the following essential steps: initiation (induction), expansion of the autophagosome membrane, maturation of the autophagosomes, and degradation, each step requiring the orchestration of several ATG genes.^4^

### Initiation/induction

Although autophagy occurs at basal levels in most cells, diverse environmental stressors and nutrient deprivation are strong inducers of this degradative pathway.^5^ The nutrient sensor mammalian target of rapamycin (mTOR) is a key negative regulator of autophagy,^6, 7^ although autophagy can also be regulated by mTOR-independent pathways.^8^ In conditions of nutrient availability, mTOR inhibits autophagy through phosphorylation and inactivation of key downstream targets, un-coordinated 51 (UNC-51)-like kinase1 (ULK1), ATG13 and focal adhesion kinase family interacting protein of 200 kD (FIP200), which form part of a complex that initiates autophagy.^9^ In contrast, the absence of nutrients, or treatment with rapamycin, inhibits mTOR, allowing ULK1 to form a complex with ATG13 and FIP200 that activates autophagy. Once autophagy is activated, it begins with the formation of the phagophore, the origin of which remains under debate, with the endoplasmic reticulum (ER), Golgi complex, mitochondria or plasma membrane via clathrin-mediated endocytosis as possible sources.^10, 11, 12, 13^ There are mainly two essential components to regulate this process. The first one is Vps34, a class III phosphatidylinositol 3-kinase, which can generate phosphatidylinositol-3-phosphate necessary for recruitment of ATG17 and ATG13 in the region of phagophore formation.^14^ Vps34 also associates with other molecules involved in autophagosome formation such as BECLIN1, p150, UV radiation resistance associated, ATG14 or autophagy/Beclin-1 regulator 1.^15, 16, 17^ The second component involved in the biogenesis of autophagosomes is ULK1, which associates with FIP200 and ATG13. Their association is known to lead to proper localization of ULK1 and stimulate its kinase activity.^18^

### Expansion of autophagosomal membranes

The elongation of phagophores depends on two ubiquitin-like conjugating systems. First is the ATG12–ATG5–ATG16L system: ATG12 is conjugated into ATG5 via ATG7 (E1-like enzyme) and ATG10 (E2-like enzyme) and then the conjugated ATG12–ATG5 complex associates with ATG16L.^19, 20, 21, 22^ The ATG12–ATG5–ATG16L complex localizes to the outer membrane of the elongating autophagosomes, but it dissociates from the membrane before autophagosome formation is completed. A recent study demonstrated that under certain stress conditions macroautophagy can occur independently of ATG5/ATG7, suggesting that there is an alternative pathway to form autophagosomes.^23^ Second is the phosphatidylethanolamine (PE)–light chain 3 (LC3) system: Microtubule-associated protein 1 LC3 (or simply LC3) is conjugated to PE. LC3 is known to promote membrane tethering.^24, 25^ Cytosolic form of LC3, LC3-I, is generated by cleavage of pro-LC by ATG4B and further processed by ATG7 and ATG3 to be conjugated to PE (LC3-II).^26^ LC3-II specifically associates with autophagosome membranes, and its levels therefore correlate with the number of autophagosomes.

### Autophagosome maturation and recycling

Complete autophagosomes fuse with endosomes or lysosomes resulting in the formation of amphisomes or autolysosomes, respectively.^27, 28^ Autophagosomes are transported to the vicinity of endosomes or lysosomes along microtubules using dynein–dynactin complex.^29^ The fusion of autophagosomes with endosomes/lysosomes requires some non-ATG components such as the endosomal sorting complex, soluble N-ethylmaleimide-sensitive factor attachment protein receptors, RAB proteins and ATPases.^30, 31, 32^ In the final step, cytoplasmic components are actually degraded in autolysosomes. Therefore, the activity of lysosomes is necessary for degradation. Deficiency of lysosomal enzymes such as cathepsin is known to induce blockage of degradation in the autophagic pathway.^33^ There is an ongoing effort to understand where and when autophagy occurs *in vivo* and how it is regulated in different tissues. To this end, transgenic mice were generated that systemically express GFP fused to LC3, serving as a marker protein for autophagosomes.^34^ This study revealed that autophagy is differentially induced by nutrient starvation in diverse tissues. In some of the tissues tested, autophagy was also found to occur actively without starvation treatments, suggesting that the extent of autophagy is organ dependent and its occurrence is not restricted to the starvation response.

## Autophagy in CNS Physiology

Neurons are highly specialized postmitotic cells, typically composed of a soma (or a cell body), a dendritic arborization and an axon. The detailed process of autophagy in such a highly differentiated and compartmentalized cell and its relevance in neuronal physiology only begin to be elucidated, as it becomes increasingly appreciated that the removal of misfolded proteins, protein aggregates and damaged organelles is of crucial importance for the proper function of neurons. Although autophagy is most frequently studied in the context of stress and pathology, recent studies indicate that in the nervous system it is a physiological process that occurs constitutively at baseline levels under normal conditions. In this section, we will review the physiological roles of autophagy in neuronal development and function. Previous studies *in vivo* and *in vitro*, using the autophagosome marker GFP-LC3, indicated that autophagosomes are very scarce in healthy neurons under nutrient-rich conditions.^34, 35^ One explanation for this scarcity could be that basal autophagy occurs at very low levels in the normal brain. Yet, another possibility is that the autophagic machinery is so efficient that autophagosomes are not accumulated in healthy neurons at detectable levels. This intriguing idea has been recently supported by a study showing that inhibition of lysosomal degradation under nutrient-rich conditions caused rapid accumulation of autophagosomes in primary cortical neurons, suggesting that autophagy constitutively occurs in neurons.^36, 37^

A recent study employed dual-colour live-cell imaging to shed light into neuron-specific mechanisms of constitutive autophagosome biogenesis in primary dorsal root ganglion and hippocampal neurons. Under basal conditions, autophagosomes are continuously generated in the axon tip, by an ordered assembly of proteins recruited with stereotypical kinetics onto the developing autophagosome. Interestingly, plasma- or mitochondrial-derived membranes were not incorporated into nascent autophagosomes in the distal axon. Rather, autophagosomes were generated at double FYVE-containing protein 1-positive subdomains of the ER, distinct from ER exit sites. Biogenesis events were enriched distally but were infrequent in dendrites, the soma or midaxon.^38^

Several neuron-specific knockouts for members of the autophagic machinery were generated to address the roles of constitutive autophagy in the mammalian brain (Table 1). In 2006, it was first demonstrated that autophagy deficiency in neurons leads to neurodegeneration.^39, 40^ The generation and analysis of the first nervous system-specific conditional knockout for ATG7, an E1-like enzyme that is essential for autophagy, revealed that while the conditional knockouts were born viable and were indistinguishable from control littermates for the first days of their life, they developed growth retardation as early as P14 and demonstrated various motor and behavioural deficits.^40^ Histological analysis revealed the widespread death of neurons in most brain areas, including the cortex, hippocampus, cerebellum and amygdala. It was previously reported that a defect in autophagy in quiescent hepatocytes leads to the accumulation of large, ubiquitin-containing inclusion bodies under nutrient-rich conditions.^41^ Similarly, autophagy-deficient neurons demonstrated an age-dependent accumulation of ubiquitin-positive inclusions, despite the fact that proteosomal integrity and function remained unaffected.

**Figure 2:**
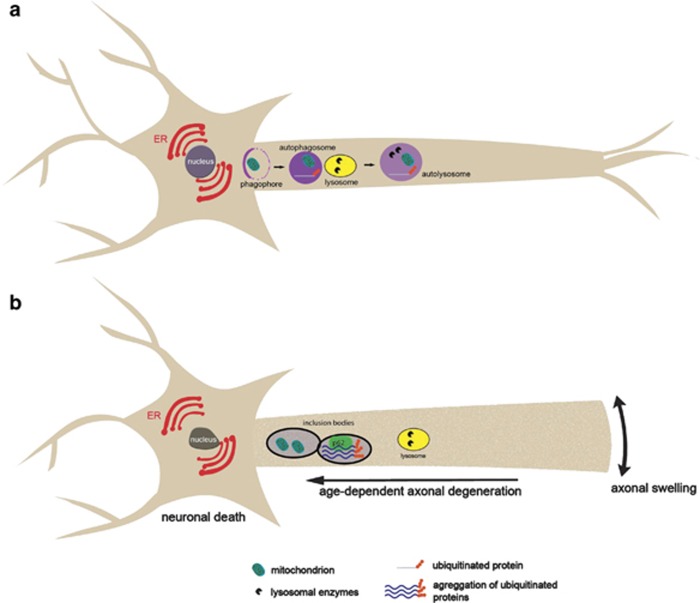
Impairment of autophagy leads to neurodegeneration. Schematic representation of (**a**) a healthy and (**b**) an autophagy-deficient neuron. Note that autophagy deficiency leads to aberrant aggregation of ubiquitinated proteins within inclusion bodies, as well as the accumulation of defective organelles such as mitochondria. Autophagy-deficient neurons display increased axonal diameter (swelling) and axonal degeneration, ultimately leading to age-dependent loss of neurons

Mice deficient for *Atg5* specifically in neural cells have also been developed and analysed. These conditional mutants developed progressive deficits in motor function that are accompanied by the accumulation of cytoplasmic inclusion bodies in neurons. In *Atg5*−/− cells, diffuse, abnormal intracellular proteins accumulate and then form aggregates and inclusions.^39^ Taken together, the results of these two studies, summarized in Figure 2, suggest that the continuous clearance of diffuse cytosolic proteins through basal autophagy is important for preventing the accumulation of abnormal proteins, which can disrupt neural function and ultimately lead to neurodegeneration. Notably, the degree of vulnerability of neurons and the formation of intracellular inclusions vary significantly among different neuron types in the mutant mice deficient in autophagy, suggesting a cell-type-specific cellular response to autophagy deficiency and a cell-type-dependent mechanism contributing to the neurotoxicity in the mutant mice.

In the models discussed above, autophagy was impaired not only in neurons but also in glial cells, yet the role of autophagy in glia and the contribution of defective glial autophagy in neurodegeneration remain poorly characterized.

Similarly, a recent study revealed that mutations in the *glucocerebrosidase* (*gba*) gene, causing Gaucher disease, the most common lysosomal storage disorder (LSD), resulted in impairment of mitophagy in neurons and astrocytes alike.^42^ Another study provided more direct evidence on the contribution of dysfunctional astrocytes in a severe LSD caused by mutations in the sulphatase modifying factor 1 (SUMF1) gene. In this study, astrocyte-specific deletion of Sumf1 *in vivo* caused severe lysosomal storage and autophagy dysfunction. Notably, dysfunction in astrocytes was sufficient to induce degeneration of cortical neurons *in vivo* in a non-cell autonomous manner.^43^ Moreover, several studies have demonstrated that astrocyte-specific overexpression of Nrf2, an antioxidant transcription factor, reduces chemical-mediated neurotoxicity modeling PD and Huntington's disease,^44, 45^ as well as genetically induced motor neuron degeneration in models of amyotrophic lateral sclerosis (ALS).^46^ A recent paper further indicates that Nrf2 in astrocytes delayed CMA and macroautophagy dysfunction observed in a mouse model of PD selectively expressing human mutant SYN (hSYN^A53T^) in neurons.^47^

Accumulating evidence suggests that changes in the metabolic signature of astrocytes underlie their response to neuroinflammation; however, the mechanisms by which pro-inflammatory stimuli induce these changes are elusive. By monitoring astrocytes following acute cortical injury, a recent study identified a differential region-specific remodelling of the astrocytic mitochondrial network in response to inflammation triggered by acute injury.^48^ Although astrocytes within the penumbra of the lesion undergo mitochondrial elongation, those located in the area invaded by pro-inflammatory cells experience transient mitochondrial fragmentation. Furthermore, maintenance of the mitochondrial architecture critically depended on the induction of autophagy. Deletion of *Atg7*, required for autophagosome formation, prevented the re-establishment of tubular mitochondria, leading to marked reactive oxygen species (ROS) accumulation and cell death. Thus these findings indicate that autophagy or specifically mitophagy is essential for regenerating astrocyte mitochondrial networks during inflammation. It is not yet clear whether neuronal degeneration in the absence of autophagy results from axonal defects; however, there is evidence implicating autophagy in axonal maintenance.^49^ For instance, ATG7 was shown to have an essential role in the maintenance of axonal homeostasis and the prevention of axonal degeneration.^50^

Likewise, in *C. elegans*, deficiency of UNC-51, encoding a serine/threonine protein kinase orthologous to yeast ATG1P and the vertebrate ULK proteins, caused disruptions in axonal membrane structures.^51^ The murine homologue is also required for neurite extension during axonal growth indicating its possible role in homeostasis of axonal membrane network.^52^ In addition, neural-specific deletion of FIP200, involved in autophagosome biogenesis, caused axonal degeneration in cerebellar neurons eventually causing their death.^53^ Thus it is possible that defects of basal autophagy affect axonal structure and function by attenuating retrograde axonal transport. In the developing nervous system, synapse elimination involves a massive loss and eventual disappearance of cellular material. This developmental reorganization causes a large majority of nascent synaptic terminals and their associated axonal branches to be removed. Because of tissue accessibility, synapse elimination has been best studied in early postnatal life at the neuromuscular junction^54^ but is also known to occur in autonomic ganglia,^55, 56^ the cerebellum,^57, 58^ the retinogeniculate system^59^ and the somatosensory system^60^ by a common mechanism of axon pruning throughout the peripheral nervous system and CNS. Retreating axon branches are pruned by a shedding process in which axonal fragments, called ‘axosomes' are engulfed by Schwann cells sheathing the retreating axon. However, the fate of these axonal remnants within the glial cells remains unclear. Studies indicate that axonal pruning is significantly delayed in mutants with lysosomal deficiency, highlighting the role of autophagic and heterophagic digestion of axonal remnants during axon pruning.

In addition, autophagy has been recently implicated in the regulation of synaptic plasticity. A study analysed transgenic mice in which macroautophagy was selectively inactivated in dopamine neurons, obtained by crossing a DAT-Cre deleter with *Atg7*-floxed mice.^61^ The analysis of these conditional knockouts was performed up to 3 months of age, and during this period no differences were observed compared with control littermates with respect to survival, motor skills and behavioural outputs. However, chronic macroautophagy deficiency in dopamine neurons resulted in increased size of axon profiles, increased evoked dopamine release and more rapid presynaptic recovery. In mice with intact macroautophagy, mTOR inhibition with rapamycin acutely increased the formation of autophagic vesicles axons, decreased the number of synaptic vesicles and depressed evoked dopamine release. However, rapamycin had no effect on evoked dopamine release and synaptic vesicles in dopamine-neuron specific macroautophagy-deficient mice. The exact substrates of autophagy in the context of regulation of presynaptic neurotransmission remain to be characterized. However, it is well accepted that disruption of mTOR signalling by rapamycin results in a reduction of late-phase LTP induced by high-frequency stimulation, while the early phase of LTP remains unaffected.^62^ Rapamycin also blocks the synaptic potentiation induced by brain-derived neurotrophic factor in hippocampal slices, demonstrating an essential role for mTOR signalling in the expression of two forms of synaptic plasticity that require novel protein synthesis. Taken together, these findings invite the speculation that these forms of synaptic plasticity may require the attenuation of constitutive autophagic activity, a hypothesis that needs to be experimentally investigated in more detail in the future.

A recent study examined the role of autophagy in adult neurogenesis. Ablation of FIP200, a gene essential for autophagy induction in mammalian cells, resulted in a progressive loss of neural stem cells (NSCs) and impairment in neuronal differentiation specifically in the postnatal brain, but not the embryonic brain, in mice. The defect in maintaining the postnatal NSC pool was caused by p53-dependent apoptotic responses and cell cycle arrest. However, the impaired neuronal differentiation was rescued by treatment with the antioxidant *N*-acetylcysteine but not by p53 inactivation. These data suggest that FIP200-mediated autophagy contributes to the maintenance and functions of NSCs by regulating the oxidative state of adult neural stem cells.^63^

The selective degradation of mitochondria, mitophagy, is also emerging as a potential modulator of dendritic growth. Collapsin response mediator protein 5 (CRMP5) has an important role in the regulation of neuronal polarity by inhibiting dendrite outgrowth at early developmental stages. A recent study demonstrates that in the brain mitophagy is induced by the translocation of CRMP5 to the inner mitochondrial membrane.^64^ It will be important in the future to understand what signals induce the translocation of CRMP5 as well as the mechanistic details that trigger mitophagy downstream this translocation event. Another study suggests that, at least in cortical neurons, mitophagy is facilitated by cardiolipin, an inner mitochondrial membrane phospholipid that becomes externalized and associated with LC3 in response to pro-mitophagic stimuli.^65^ Recent evidence suggests that, in mammals, FOXO factors activate mitophagy at the transcriptional level by increasing the expression of mitochondrial E3 ubiquitin ligases, which in turn target mitochondria to the phagophore for degradation, and this may be a protective mechanism in mammalian models of Parkinson's disease.^66^ In addition, autophagy was shown to be a key process for the regulation of the visual cycle.^67^ RPE cells have numerous important functions, among which is the phagocytic activity, as well as the ability to perform the biochemical reactions for the generation of the chromophore that is transferred to rods and cones and, upon light exposure, is responsible for initiating the phototransduction signalling cascade. Photoreceptor cells continuously renew their outer segments, in a process regulated by circadian rhythms. Outer segment tips are shed daily in the early morning, followed by a burst of phagocytosis by RPE cells to rapidly clear the retina of the debris. Disruption of RPE phagocytosis is causal to severe retinal pathologies, such as retinitis pigmentosa and rod/cone dystrophies. This study provides support for a link between a non-canonical form of autophagy, phagocytosis of photoreceptor outer segments and chromophore regeneration within the RPE, which is essential to the visual cycle. This non-canonical form, termed LC3-associated phagocytosis, occurs by formation of typical phagolysosomes and is activated upon uptake of extracellular cells, including apoptotic, necrotic and necroptotic cells.^68^

Another intriguing role of autophagy in the context of normal brain physiology is in the regulation of food intake and energy balance.^69^ The hypothalamic arcuate nucleus consists of neurochemically discrete and functionally antagonistic neurons, including agouti-related peptide (AgRP) and pro-opiomelanocortin neurons^70^ that form a focal point for the integration of nutritional and metabolic cues, central and peripheral neural afferents and action of adiposity hormones, such as leptin and insulin. This study demonstrates a role for autophagy in hypothalamic AgRP neurons in the regulation of food intake and energy balance. Starvation-induced hypothalamic autophagy is shown to mobilize neuron-intrinsic lipids to generate endogenous free fatty acids, which in turn regulate AgRP levels. The functional consequences of inhibiting autophagy are the failure to upregulate AgRP in response to starvation and constitutive increases in hypothalamic levels of pro-opiomelanocortin and its cleavage product α-melanocyte-stimulating hormone that typically contribute to a lean phenotype. Therefore, a new conceptual framework is proposed where autophagy-regulated lipid metabolism within hypothalamic neurons modulates neuropeptide levels to regulate food intake and energy homeostasis.

## Autophagy in CNS Pathologies of Developmental Origin

Impaired autophagy has been implicated in a number of neurodevelopmental disorders. Sustained deregulation of the autophagic flux generates an ‘autophagic stress' in neurons, interfering with their normal function from the onset of their development. We will discuss some examples below. LSDs comprise nearly 60 different inherited disorders, caused by the inability of the lysosomal system to degrade specific metabolites, resulting in abnormal storage/accumulation within the lysosome. As a consequence, many tissues and organs are affected, with the early onset neurodegeneration within the CNS predominating. Emerging data identify autophagy dysfunction in neurons as a major component of the phenotype.^71^ Moreover, recent studies indicate that lysosomal/autophagic dysfunction in astrocytes is an important component of neurodegeneration in LSDs.^43, 72^

Niemann–Pick type C (NPC) is a LSD caused by mutations in either the *Npc1* or *Npc2* gene,^73, 74^ the functional loss of which leads to the accumulation of unesterified cholesterol and glycosphingolipids in late endosomes and lysosomes. NPC1-deficient brains are marked by neuronal loss^75, 76^and severe neurological symptoms. There is strong evidence that neurological symptoms arise as a result of impaired autophagic flux. Two recent studies indicate that while the production of autophagosomes is enhanced, progression of the autophagic process is stalled leading to the accumulation of defective mitochondria in NPC1-deficient neurons,^77, 78^ raising the possibility that mitophagy is specifically a responsible phenotype.

Niemann–Pick disease type A (NPA), which is caused by loss of function mutations in the *acid sphingomyelinase* (*Asm*) gene, is another LSD leading to neurodegeneration. A recent study demonstrated that autophagolysosomes containing un-degraded molecules accumulate in neurons of *Asm* knockout mice and in fibroblasts from NPA patients, due to inefficient autophago-lysosomal clearance. These defects can be induced in control cells by addition of sphingomyelin and, conversely, they can be reverted in ASM-deficient cells by lowering the levels of sphingomyelin. These findings suggest a role for sphingomyelin in autophagy modulation, opening new perspectives for therapeutic interventions.^79^

## Autophagy and Neurodegenerative Diseases

### Interplay between autophagy and necrosis

Growing evidence has shown that cell death mechanisms, rather than being discrete, are activated by common pathways. Although apoptosis is involved in the physiological development of the nervous system, necrotic cell death is considered pathological as it includes an inflammatory response. Necrosis is a type of unprogrammed cell death brought upon damage or pathological conditions. Autophagy is an essential homeostatic cell response mechanism that is implicated in both types of cell death. Understanding the intricate crosstalk between autophagy and necrosis is essential, as this process, on the one hand, is required for necrosis while, on the other, can act as a pro-survival mechanism to inhibit cell demise.

Autophagy, which can be triggered by starvation, has been reported to suppress necroptosis, which is a type of programmed necrotic cell death in response to tumor necrosis factor α, in mammalian cell lines, such as lymphocytes and cancer cells.^80, 81^ Moreover, a general caspase inhibitor, zVAD, which induces necroptosis, simultaneously blocks apoptosis and autophagy by inhibiting lysosomal cathepsins. Accordingly, under starvation conditions, when autophagy is induced, zVAD-induced necroptosis is suppressed.^82^ Autophagy can also promote necroptosis under acute pathological conditions.^83^ At the molecular level, it has been shown that an NAD^+^-dependent protein deacetylase, SIRT1, which affects stress resistance and aging, assembles with the autophagic machinery to promote autophagy.^84^ Another member of the sirtuin family, SIRT2, permits the interaction of the two kinases required for necroptosis, receptor interacting protein 1 (RIP1) and RIP3.^85^ However, delineation of the regulatory mechanisms and actual inter-relationship of autophagy and necroptosis remains to be elucidated.

Extreme stress such as oxidative stress and subsequent ROS production triggers the death-associated protein kinase/protein kinase D (PKD) pathway. This pathway can in turn either induce autophagy through Beclin-1 phosphorylation or necrosis, through PKD phosphorylation.^86, 87^ The importance of fine-tuning between necrosis and autophagy is accentuated in the case of poly(ADP-ribose) polymerase 1 (PARP1)-mediated necrosis. PARP1, depending on the triggering stress, can lead to DNA damage response or cell death. On the one hand, PARP1 activation can cause NAD+ and adenosine tri-phosphate (ATP) depletion, which would in turn make the cells prone to necrotic cell death by its cleavage by lysosomal cathepsins while blocking apoptosis, which requires energy.^88, 89^ On the other hand, DNA damage-induced PARP1 activation can deplete ATP and promote autophagy by interacting with the AMP-activated protein kinase α signalling pathway, which inhibits the mTOR pathway. Thus PARP1 could be characterized as a sensor under acute cellular stress, which can either promote cell survival through autophagy or trigger necrotic cell death.^90^ Interestingly, there is emerging evidence of a new cell death mechanism after exposure to palmitoleic acid, termed ‘liponecrosis' that shares only some characteristics of necrotic cell death. Induction of a selective type of macroautophagy, mitophagy, which is the degradation of damaged mitochondria, protected against liponecrotic cell death.^91^

### Neurodegenerative pathologies

As discussed above, neurons are specifically sensitive to dysfunction of the autophagic machinery. First, upon excessive increase of dysfunctional structures, neuronal necrosis and excitetoxicity will occur.^92^ The importance of macroautophagy in neuronal homeostasis and function becomes more notable when observing the amount of autophagosomes in neurons in neurodegenerative disease.^39, 93^ Thus the inter-relationship between autophagy and neurodegeneration has attracted great interest over the past years. *Clcn7* gene mutations, which encodes for the CIC-7chloride channel, causes LSD.^94^ This protein, among others, consists of a *β*-subunit, osteopetrosis-associated transmembrane protein 1 (Ostm1) membrane protein, which is required for its normal function and interaction with the lysosomes.^95^ *Ostm1* deficiency induces severe neurodegeneration after reactive gliosis and inflammation due to defect in the autophagic machinery that caused abnormal accumulation of autophagosomes.^96^

Dysregulation of autophagy is prominent at distinct stages in Parkinson's disease, where *α*-synuclein aggregates in ‘Lewy bodies'.^97^ A newly revealed autophagy step that is disturbed in this disease is endosomal protein sorting to the Golgi, by mutating a retromer protein, VPS35, which then inhibits WASH complex trafficking to endosomes and ultimately autophagosome formation.^98^ The autophagic machinery malfunction can extend its detrimental effects to neighbouring neurons, as it induces exocytosis and intercellular transfer of the aggregates.^99^ Of note, disruption in mitophagy has emerged as a major cause in Parkinson's disease. In particular, mutated forms of PINK1 and PARKIN, which have been implicated in Parkinson's disease, are essential for mitochondrial biogenesis and recycling. Therefore, mitochondrial quality control by mitophagy is disrupted in this neurodegenerative disease.^100^ Similarly, in polyglutamine disorders, protein aggregates form. One characteristic example is that of spinocerebellar ataxia 7 (SCA7), where ATAXIN7, a protein containing long polyglutamine tract, is found to be located in autophagosomes. Interestingly enough, these results were also confirmed in the cerebellum and cerebral cortex in human SCA7 patients as well.^101^

Another instance where multiple defects in neuronal intracellular lysosomal degradation result in neurodegenerative disease progression is in the case of ALS.^102^ Surprisingly, a recent study identifies BECLIN1, a major player in the autophagic initiation process, as a factor that promotes ALS by interacting with the major mutated gene implicated in this disease, *sod1* (superoxide dismutase 1).^103^ On the contrary, aggregation of plaques of amyloid-*β* tau tangles, which are the major cause of Alzheimer's disease (AD), is increased upon downregulation of BECLIN1.^104^ Related to this, a recent study indicated that BECLIN1 is required for efficient phagocytosis *in vitro* and in mouse brains and that BECLIN1-mediated impairments in phagocytosis are associated with dysfunctional recruitment of retromer to phagosomal membranes, reduced retromer levels and impaired recycling of phagocytic receptors CD36 and Trem2. Interestingly, microglia isolated from human AD brains showed significantly reduced BECLIN1 and retromer protein levels.^105^ In line with this, the AP2/PICALM complex was recently shown to interact with LC3 as well as the amyloid precursor, linking the macroautophagy and amyloid-*β* degradation.^106^ Interestingly, in this and other forms of dementia, the transcription factor REST, which negatively regulates the expression of cell death genes, is lost from the nucleus. Instead, it was located together with pathological misfolded proteins in autophagosomes.^107^ Thus discerning the autophagy process role in a neurodegenerative disease context could prove quite complex.

An alternative form of autophagy, LC3-associated phagocytosis (LAP), mentioned earlier, is involved in new photoreceptor generation by degradation of photoreceptor outer segments while recycling retinoic acid, as discussed above.^67^ Another study has underlined that a defect in the autophagic machinery elicits a strong inflammatory response.^108^ Taken together, one could infer that necrotic cells that cannot be properly be phagocytosed and lysed by LAP trigger a strong inflammatory response that could, in turn, cause further cell death and neurodegeneration.

## Conclusions and Outlook

Accumulating evidence indicates that maintaining a balanced autophagic flux is essential in neuronal physiology. Impairment of any step of the autophagic pathway in neurons generally results in aberrant neuronal homeostasis, which manifests itself with axonal defects and culminates in neuronal degeneration. However, different subtypes of neurons in the brain exhibit different degrees of dependence on the autophagic pathway and are differentially vulnerable to perturbations of the autophagic flux. Future studies should aim to investigate the cellular background explaining such differences, as well as how they may relate to the selective vulnerabilities of specific populations of neurons in different diseases. In addition to the general tasks autophagy performs in neurons, the autophagic machinery appears to be essential also for highly specialized circuits and processes, such as the control of the visual cycle and of satiety.

Autophagy is also implicated in a number of neurodevelopmental and neurodegenerative disorders, either as a cause or as a consequence of the underlying aetiology. To better comprehend the involvement of autophagy in the maintenance and pathogenesis of the CNS, a great deal of effort should be invested in understanding (a) the upstream signals that regulate the autophagic machinery in neurons under physiological conditions and (b) where and when selective types of autophagy occur in neurons during normal development and physiology.

## Figures and Tables

**Table 1:** Summary of phenotypes resulting from genetic ablations of autophagic machinery components in neurons

**Genetic ablation of autophagic component**	**Phenotype**	**Reference**
*atg7* (CNS-specific ablation)	Motor deficits Behavioural defects Loss of axonal homeostasis Early onset neurodegeneration	^40, 50^
*atg5* (CNS-specific ablation)	Motor deficits Early onset neurodegeneration	^39^
*FIP200* (CNS-specific ablation)	Degeneration of cerebellar Purkinje neurons	^53^
*atg7* (dopaminergic neuron-specific ablation)	Presynaptic defects: Increased axon profiles Increased evoked dopamine release Increased presynaptic recovery	^61^
*atg5* (RPE-specific ablation)	Reduced recycling of photoreceptor outer segments Reduced chromophore regeneration	^67^
*atg7* (AgRP neuron- specific ablation)	Failure to upregulate AgRP Increased POMC	^69^
*ambra1* (heterozygotes, germline mutation)	Autism-like phenotype in females only: Compromised communication and social interactions Enhanced repetitive behaviours Impaired cognitive flexibility	^109^

## Abbreviations

AgRP: agouti-related peptide
ALS: amyotrophic lateral sclerosis
ATG: autophagy gene
ATP: adenosine tri-phosphate
CNS: central nervous system
CRMP5: collapsin response mediator protein 5
ER: endoplasmic reticulum
ESCRT: endosomal sorting complex required for transport
FIP200: focal adhesion kinase family interacting protein of 200 kD
LAP: LC3-associated phagocytosis
LC3: light chain 3
LSD: lysosomal storage disorder
LTP: long-term potentiation
MAP1: microtubule-associated protein 1
mTOR: mammalian target of rapamycin
NPC: Niemann–Pick type C
OSTM1: osteopetrosis-associated transmembrane protein 1
PARP1: poly (ADP-ribose) polymerase 1
PE: phosphatidylethanolamine
PKD: protein kinase D
RIP1: receptor interacting protein 1
ROS: reactive oxygen species
RPE: retinal pigmented epithelium
SCA7: spinocerebellar ataxia 7
ULK1: UNC-51-like kinase 1
UNC-51: un-coordinated 51
UVRAG: UV radiation resistance associated
